# Photobiomodulation Therapy Restores IL-10 Secretion in a Murine Model of Chronic Asthma: Relevance to the Population of CD4^+^CD25^+^Foxp3^+^ Cells in Lung

**DOI:** 10.3389/fimmu.2021.789426

**Published:** 2022-02-02

**Authors:** Aurileia Aparecida de Brito, Tawany Gonçalves Santos, Karine Zanella Herculano, Cintia Estefano-Alves, Cristiano Rodrigo de Alvarenga Nascimento, Nicole Cristine Rigonato-Oliveira, Maria Cristina Chavantes, Flávio Aimbire, Renata Kelly da Palma, Ana Paula Ligeiro de Oliveira

**Affiliations:** ^1^ Department of Research, Development and Innovation, Innovative Health System Health Management (IHS Medicine and Technology), São Paulo, Brazil; ^2^ Post-Graduate Program in Biophotonics Applied to Health Sciences, University Nove de Julho (UNINOVE), São Paulo, Brazil; ^3^ Post-Graduate Program in Medicine, University Nove de Julho (UNINOVE), São Paulo, Brazil; ^4^ Translational Medicine, Federal University of São Paulo—UNIFESP, São José dos Campos, Brazil; ^5^ Department of Surgery, School of Veterinary Medicine and Animal Sciences, University of São Paulo, São Paulo, Brazil; ^6^ Facultad de Ciencias Experimentales, Universidad Francisco de Vitoria, Madrid, Spain; ^7^ Human Movement and Rehabilitation, Post-Graduate Program Medical School, Evangelic University of Anápolis—UniEVANGELICA, Anápolis, Brazil

**Keywords:** bronchial asthma, airway inflammation, lung mechanics, Treg cells, IL-10, mice

## Abstract

It is largely known that photobiomodulation (PBM) has beneficial effects on allergic pulmonary inflammation. Our previous study showed an anti-inflammatory effect of the PBM in an acute experimental model of asthma, and we see that this mechanism is partly dependent on IL-10. However, it remains unclear whether the activation of regulatory T cells is mediated by PBM in a chronic experimental model of asthma. In this sense, the objective of this study was to verify the anti-inflammatory role of the PBM in the pulmonary inflammatory response in a chronic experimental asthma model. The protocol used for asthma induction was the administration of OVA subcutaneously (days 0 and 14) and intranasally (3 times/week, for 5 weeks). On day 50, the animals were sacrificed for the evaluation of the different parameters. The PBM used was the diode, with a wavelength of 660 nm, a power of 100 mW, and 5 J for 50 s/point, in three different application points. Our results showed that PBM decreases macrophages, neutrophils, and lymphocytes in the bronchoalveolar lavage fluid (BALF). Moreover, PBM decreased the release of cytokines by the lung, mucus, and collagen in the airways and pulmonary mechanics. When we analyzed the percentage of Treg cells in the group irradiated with laser, we verified an increase in these cells, as well as the release of IL-10 in the BALF. Therefore, we conclude that the use of PBM therapy in chronic airway inflammation attenuated the inflammatory process, as well as the pulmonary functional and structural parameters, probably due to an increase in Treg cells.

## Introduction

Bronchial asthma is among the most common chronic respiratory diseases (1–3). This disease generates a pulmonary inflammatory response that is mitigated by non-curative treatments that have serious side effects (4, 5). A lot of research has been invested in the search for new drugs to treat this condition. Although drug therapy is classically the first option for the treatment of chronic lung diseases, a growing number of studies have shown that photobiomodulation (PBM) therapy can be a low-cost and effective option in aiding the treatment of inflammatory and fibrotic diseases in general (6–11). In this sense, several studies have already demonstrated the effectiveness of PBM therapy in experimental models of acute lung injury (12), asthma (13, 14), COPD (15), and idiopathic pulmonary fibrosis (16).

These studies demonstrate in general that the transcutaneous application of laser at low intensity reaches the lungs and positively interferes in the inflammatory/immunological processes of acute lung injury, whether induced by the administration of lipopolysaccharide (LPS) and ovalbumin (OVA), or by intestinal ischemia and reperfusion (17–19). They also demonstrated that, in part, these anti-inflammatory effects of PBM are due to the increase in IL-10 in the lung (20). Therefore, it seems reasonable to us that the success of this therapy depends on a greater understanding of biological processes associated with its anti-inflammatory effects, both in the treatment of lung diseases and in the treatment of other diseases.

Our previous study showed an anti-inflammatory effect of PBM in an acute experimental model of asthma, and we showed that this mechanism is partly dependent on IL-10 (14). However, the activation of regulatory T cells is mediated by PBM in an experimental model of chronic asthma.

Therefore, the present study demonstrates that laser irradiation in animals after antigenic challenge in an experimental asthma model reduced inflammation and lung remodeling, mucus, and collagen production, as well as lung mechanics. In addition, it is worth highlighting the increase in Treg cells (CD4^+^CD25^+^Foxp3^+^) with a consequent increase in the release of the anti-inflammatory cytokine IL-10, contributing to the reduction of allergic pulmonary inflammation. Thus, the objective of this study was to verify the anti-inflammatory role of the PBM in the pulmonary inflammatory response in a chronic experimental asthma model.

## Materials and Methods

### Animals

The animals were obtained from the breeding facility of Universidade Nove de Julho and kept under controlled conditions of humidity (50%–60%), light (12-h light/12-h dark), and temperature (22°C–25°C) in the Experimental vivarium of the Nove de Julho University. Approximately 100 male mice (Balb/C), males, weighing approximately 20–25 g, were used for the project (we will divide this total of animals, so that the experimental protocol is carried out in two different sets). The experiment was approved by the Ethics Committee on Animal Research from the Federal University of São Paulo (protocol number 9938270115).

### Chronic Allergic Pulmonary Inflammation

For induction of chronic allergic pulmonary inflammation using ovalbumin (OVA), the animals were sensitized with a subcutaneous (s.c.) injection of 4 µg of OVA (Sigma) together with Alum gel solution on days 0 and 14. From day 21 onwards, the animals were submitted to orotracheal challenge with 10 µg of OVA, 3 times a week for 5 weeks. For this procedure, the animals were subjected to adequate immobilization in a position that allows appropriate access to the route of administration, for application of anesthesia with intramuscular (i.m.) injection of 2% xylazine (0.06 ml/100 g) + 10% ketamine (0.08 ml/100 g). Minutes later, OVA instillation was performed.

### Photobiomodulation Therapy

The animals were irradiated with a diode laser, with a power of 100 mW and a wavelength of 660 nm, irradiating an area of 0.045 cm^2^ with an energy density of 5 J. One hour after each challenge (Group OVA + LBI), the animals received (50 s) punctual application in three regions: one below the trachea, and the other two in each lung lobe (right and left).

### Experimental Groups

All mice were placed in a common box and divided randomly into 3 groups containing seven animals each: (1) Basal group, which consisted of non-manipulated mice; (2) OVA group, which consisted of mice sensitized and challenged with OVA; and (3) OVA + PBM: mice sensitized and challenged with OVA and submitted to PBM therapy. For euthanasia, all experimental groups, including the Basal group, were anesthetized with 100 mg/kg of ketamine and 10 mg/kg of xylazine intraperitoneal.

### Quantification of Lung Inflammation in Bronchoalveolar Lavage Fluid

After anesthesia with ketamine (100 mg/kg) and xylazine (10 mg/kg), the animals were exsanguinated and blood was collected, tracheostomized, and cannulated, and the lungs were washed with 3 × 0.5 ml of phosphate buffered saline (PBS). The recovered lavage volume was centrifuged at 1,600 rpm at 4°C for 5 min. The supernatant will be stored at −70°C for cytokine analysis by ELISA. The cell button was resuspended in 1 ml of phosphate-buffered saline (PBS) and used for BAL cell determination performed by Neubauer chamber (total cells) and to make the cytospin slide, stained with Instant Prov (differential cells).

### Evaluation of Cytokine and Inflammatory Mediators Levels in BALF

The levels of IL-1, TNF-, IL-4, IL-5, IL-10, IL-13, and LTB_4_ in BALF were evaluated using the Biolegends kit and R&D Systems.

### Analysis of Airway Remodeling

In order to assess the effects of PBM therapy on the volume ratio of collagen fibers and mucus production in the airway wall. For this purpose, the left lung was collected, fixed in 10% formalin, and embedded in paraffin; 4-μm-thick cuts were made and the slides were stained with Picrosirius for detection of collagen fibers and with Periodic Acid Schiff (PAS) for detection of mucus. Quantitative analysis was performed using the morphometric technique. Morphological parameters were evaluated using Image Pro Plus software (version 4.5, NIH, Maryland, USA). Five airways of each animal were analyzed.

### Mucus Production

Mucus production in the airway was quantified by the morphometric method. Morphological parameters were evaluated through Image Pro Plus software (version 4.5, NIH, Maryland, USA). The measurement was performed in 5 complete airways of each animal at 1,000 × magnification. First, the area of interest of the bronchial epithelium in mm^2^ was selected, then the mucus area (mm^2^) was calculated:


$$\frac{\text{Mucus area value }({\text{mm}}^{2})\times 1000}{\text{Area of interest }({\text{mm}}^{2})}$$

Therefore, the unit of mucus quantification in the airways is in mm^2^/mm^2^.

### Assessment of Lung Mechanic

The animals were anesthetized with xylazine and ketamine (i.p.) at a dose of 0.004 mg/g and placed on the surgical table, where a small longitudinal incision was made in the anterior region of the animal’s neck. Adjacent tissues were divulged until the trachea was exposed, at which point a transverse incision was made between two fibrous rings so that a tracheostomy cannula for small animals could be introduced. The animal was then taken to the recording system, where the tracheostomy cannula was connected to a pneumotachograph to measure tracheal flow by pressure drop sensitivity through a pneumotachograph with a differential pressure transducer (Hans Rudolph Inc., Shawnee, USA). Tracheal pressure was checked by connecting a pressure transducer to the lateral port located between the pneumotachograph and the cannula. The pneumotachograph inlet was connected to a Y-piece of a volumetric mechanical ventilator (MV215, Montevideo, UY) designed for artificial ventilation of rodents. The lungs were subjected to conventional mechanical ventilation in two different ways, open (lung) and closed chest (respiratory system). The parameters of ventilation used were a quasi-sinusoidal flow pattern with a tidal volume of 10 ml/kg of mouse body weight, a frequency of 100 breaths/min, and a positive end expiratory pressure of 2 cm H_2_O. Flow and pressure signals from the transducers were analogically low-pass filtered, sampled, and stored for subsequent analysis. Using these parameters, the static (Est) and dynamic elastance (Edyn) were obtained and analyzed. The results were expressed in cm H_2_O ml^−1^.

### Identification of Recruited Lung Cells by Flow Cytometry

Lung tissue was fragmented into small pieces and incubated with collagenase IV and deoxyribonuclease I (DNAse) (Sigma) 2 mg/ml and 1 mg/ml, respectively, for 30 min at 37°C under continuous agitation. After this period, we added Hank`s balanced solution (HBSS) plus EDTA to slow down the digestion of the material. The lung fragments were massed and filtered through a 40-μm sieve and the contents were centrifuged at 1,500 rpm for 10 min and then resuspended in PBS buffer. The cells were incubated for 20 min at 4°C. After the incubation period, the samples were washed with PBS containing 0.01% BSA and sodium azide and resuspended in 200 μl of the same buffer. The samples were acquired in the BD Accuri flow cytometer and analyzed in the CSampler software (Becton Dickinson - BD^®^, East Rutherford, NJ, USA). After the incubation period, the samples were washed with PBS containing 3% fetal bovine serum (FBS) and resuspended in 300 μl of the same buffer. After two washes with Permwash, the samples were acquired in a flow cytometer.

### Cell Phenotyping

Phenotyping analysis was performed for Treg cells with anti-CD4 FITC and anti-CD25 PE and with the transcription factor [anti-Foxp3 Percp, as well as characterization of IL-10 (anti-IL-10 APC) (Becton Dickinson - BD^®^, East Rutherford, NJ, USA]. The cells were incubated for 20 min at 4°C. After the incubation period, the samples were washed with PBS containing 0.01% BSA and sodium azide and resuspended in 200 μl of the same buffer. The samples were acquired in the BD Accuri flow cytometer and analyzed in the CSampler software (Becton Dickinson - BD^®^, East Rutherford, NJ, USA). After the incubation period, the samples were washed with PBS containing 3% fetal bovine serum (FBS) and resuspended in 300 μl of the same buffer. After two washes with Permwash, the samples were acquired in a flow cytometer.

To evaluate the expression of surface molecules, the cells obtained were incubated with 1:100 of eBioscienc® anti-CD16/32 monoclonal antibody for thirty minutes at 4oC to block Fc receptors. Then, the cells were incubated with fluorochrome-conjugated monoclonal antibodies (FITC, PE, PercP or APC) specific for the molecules of interest for 30 minutes also at 4oC. The following monoclonal antibodies (Biolegend®) were used: anti-CD3 (0.5μg/106 cells), anti-CD4 (0.5μg/106 cells), anti-CD25 (0.5μg/106 cells), anti-Foxp3 ( 0.5μg/106 cells), anti-IL10 (0.5μg/106 cells). Samples were acquired on a FACS Accuri flow cytometer (Becton ∓ Dickinson, Mountain View, CA). Results refer to the use of 5-6 mice in each experimental group. Thus, 10,000 events were acquired from each sample. Representative MFI histograms were obtained for animals in the Basal, OVA and OVA + PBM groups. Data are representative of one animal from each group.

### Statistical Analysis

Data were analyzed using SigmaStat 3.1 software (USA). Data with parametric distribution were submitted to the one-way ANOVA test followed by the Newman-Keuls test for comparison between groups. Significance levels were adjusted to 5% (*p* < 0.05). The graphs will be prepared using the GraphPad Prism 3.1 software (USA). Significance levels were adjusted to 5% (*p* < 0.05). The graphs will be prepared using the GraphPad Prism 3.1 software (California, USA).

## Results

### PBM Reduces Leukocytes Evaluated in BAL

**Figure 1:**
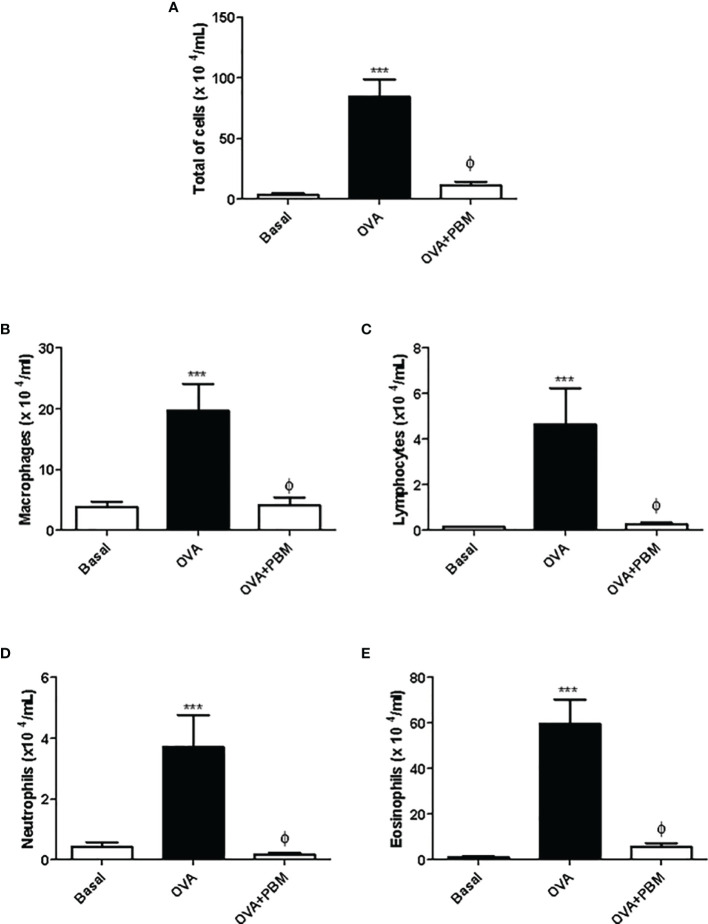
Photobiomodulation on the influx of inflammatory cells to the lung. In **(A)**, the total number of cells, **(B)** macrophages, **(C)** lymphocytes, **(D)** neutrophils, and **(E)** eosinophils recovered in the bronchoalveolar lavage (BAL) 24 h after the last challenge. Basal group *n* = 5; OVA group *n* = 7; PBM group *n* = 5; OVA+ PBM *n* = 8. Two independent sets of experiments were carried out. Data represent mean ± standard error (SEM). ***p < 0.001 in relation to the Basal group. ^ϕ^ *p* < 0.001 in relation to the OVA group.

The results showed a significant increase in the total influx of leukocytes (**Figure 1A**), as well as in the number of macrophages, lymphocytes, neutrophils, and eosinophils (**Figures 1B–E**) recovered in BAL in the OVA group when compared to the Basal group. On the other hand, PBM therapy in the OVA group reduced all leukocyte types in BAL when compared to the OVA group.

### PBM Reduces Quantification of Cytokines Pro-Inflammatory in the BAL

**Figure 2:**
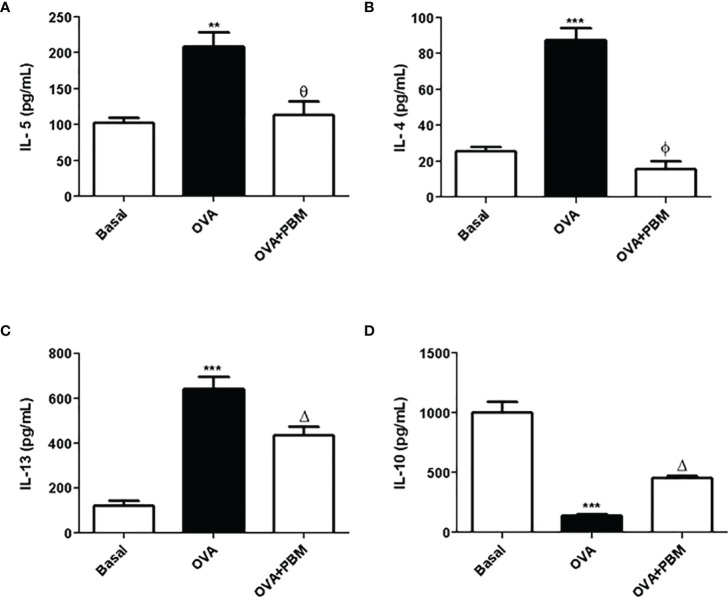
Effect of photobiomodulation on BAL cytokine. In **(A)** IL-5, **(B)** IL-4, **(C)** IL13, and **(D)** IL-10 in the BAL supernatant. Basal group, *n* = 5; OVA group, *n* = 7; PBM group, *n* = 5; OVA+ PBM, *n* = 8. Two independent sets of experiments were carried out. Data represent mean ± standard error (SEM). **p < 0.01 and ***p < 0.001 in relation to the Basal group; ^Δ^ *p* < 0.05; ^θ^ *p* < 0.01; ^ϕ^ *p* < 0.001 in relation to the OVA group.

The results showed a significant increase in the levels of the pro-inflammatory cytokines IL-5 (**Figure 2A**), IL-4 (**Figure 2B**), and IL-13 (**Figure 2C**) and a reduction of IL-10 (**Figure 2D**) in the supernatant of BAL, in the asthmatic group (OVA) when compared to the Basal group. On the other hand, there was an increase in the anti-inflammatory cytokine IL-10 (**Figure 2D**) in the asthmatic group submitted to PBM when compared to the OVA group. When we evaluated the OVA group submitted to PBM therapy, we observed a significant reduction of IL-4, IL-5, and IL-13 (**Figures 2A–C**) compared to the OVA group.

### PBM Reduces Quantification of Inflammatory Mediators in the BAL

**Figure 3:**
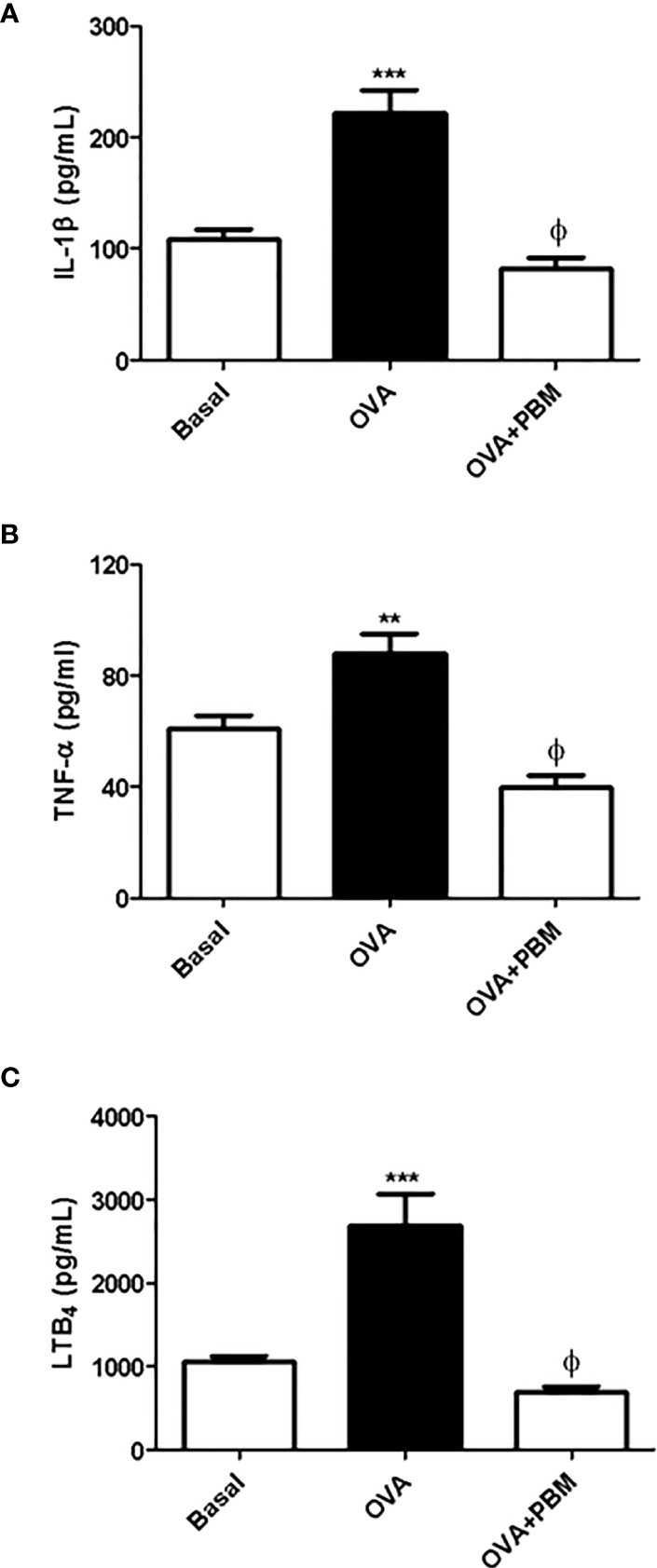
Effect of photobiomodulation on BAL inflammatory mediators levels. Quantification of IL1-β **(A)**, TNF-α **(B)**, and LTB_4_ **(C)** in BAL supernatant. Basal group, *n* = 5; OVA group, *n* = 7; PBM group, *n* = 5; OVA+ PBM, *n* = 8. Two independent sets of experiments were carried out. Data represent mean ± standard error (SEM). **p < 0.01; ***p < 0.001 in relation to the Basal group. ^ϕ^ *p* < 0.001 in relation to the OVA group.

The results showed a significant increase in the levels of IL-1β (**Figure 3A**), TNF-α (**Figure 3B**), and LTB_4_ (**Figure 3C**) in the supernatant of BAL in the OVA group, when compared to the Basal group. On the other hand, there was a reduction in all mediator levels in the supernatant of BAL (**Figure 3**) in the OVA group submitted to PBM when compared to the OVA group.

### PBM Reduces Mucus in the Airways

**Figure 4:**
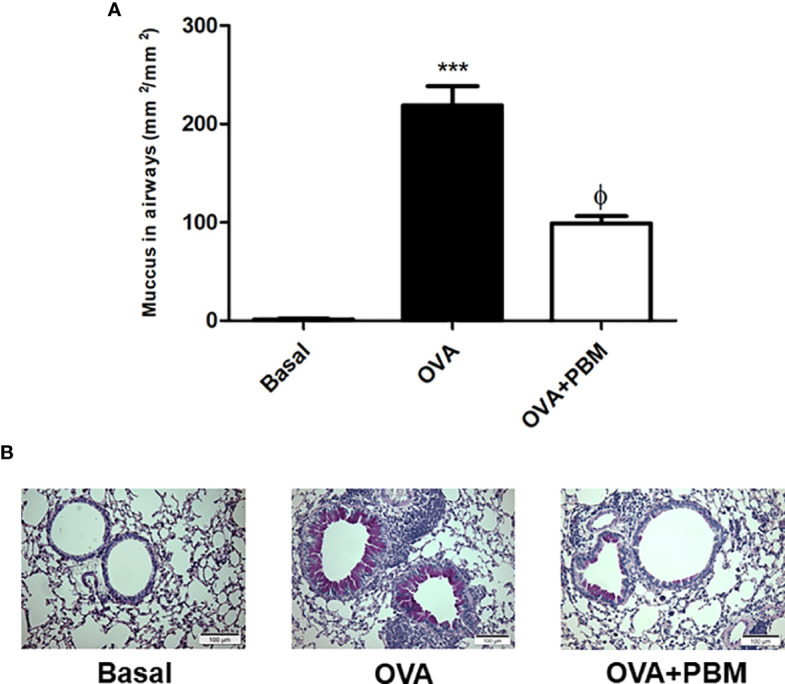
Effect of photobiomodulation on airway mucus production. Neutral mucin-producing goblet cells were stained with PAS or mucus density was evaluated in the bronchial region between epithelial basement membrane and luminal cell membrane (lumen). Quantification of mucus production in the airways **(A)**.The photomicrographs represent all the groups studied **(B)**. Basal group, n = 5; OVA group, n = 7; PBM group, n = 5; OVA+ PBM, n = 8. Two independent sets of experiments were carried out. Data represent mean ± standard error (SEM). ***p < 0.001 in relation to the Basal group. fp < 0.001 in relation to the OVA group. ^ϕ^p < 0.001 in relation to the OVA group.

In **Figure 4**, we observed a significant increase in mucus deposition in the asthmatic group (OVA) when compared to the Basal group. When we compared the OVA group that was submitted to PBM, we observed a significant effect on the reduction of mucus production in the airways (**Figure 4A**) when compared to the OVA group. The photomicrographs represent all the groups studied (**Figure 4B**).

### PBM Reduces Collagen in the Airways

**Figure 5:**
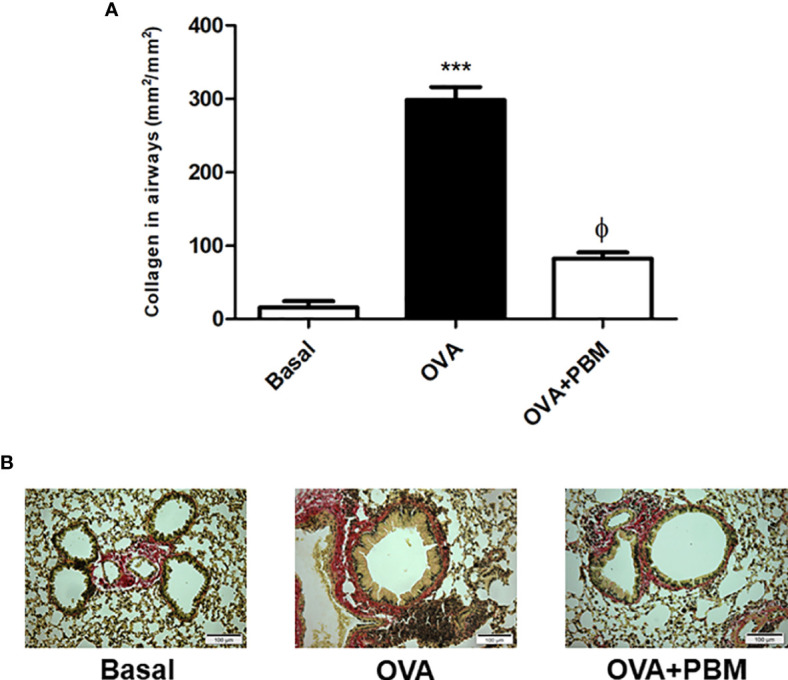
Effect of photobiomodulation on airway collagen deposition. Quantification of collagen fiber deposition in the airways **(A)** The photomicrographs represent all the groups studied **(B)**. Basal group, n = 5; OVA group, n = 7; PBM, group n = 5; OVA+ PBM, n = 8. Two independent sets of experiments were carried out. Data represent mean ± standard error (EMP). ***p < 0.001 in relation to the Basal group. fp < 0.001 in relation to the OVA group. ^ϕ^p < 0.001 in relation to the OVA group.

The results related to the quantification of collagen in the airways are presented in **Figure 5**. We found a significant increase in collagen deposition in the asthmatic group (OVA) when compared to the Basal group. When we compared the OVA group that was submitted to PBM (OVA + PBM), we observed a significant effect on the reduction of collagen fiber deposition in the airways (**Figure 5A**) when compared to the OVA group. The photomicrographs represent all groups evaluated (**Figure 5B**).

### PBM Reduces Lung Mechanics

**Figure 6:**
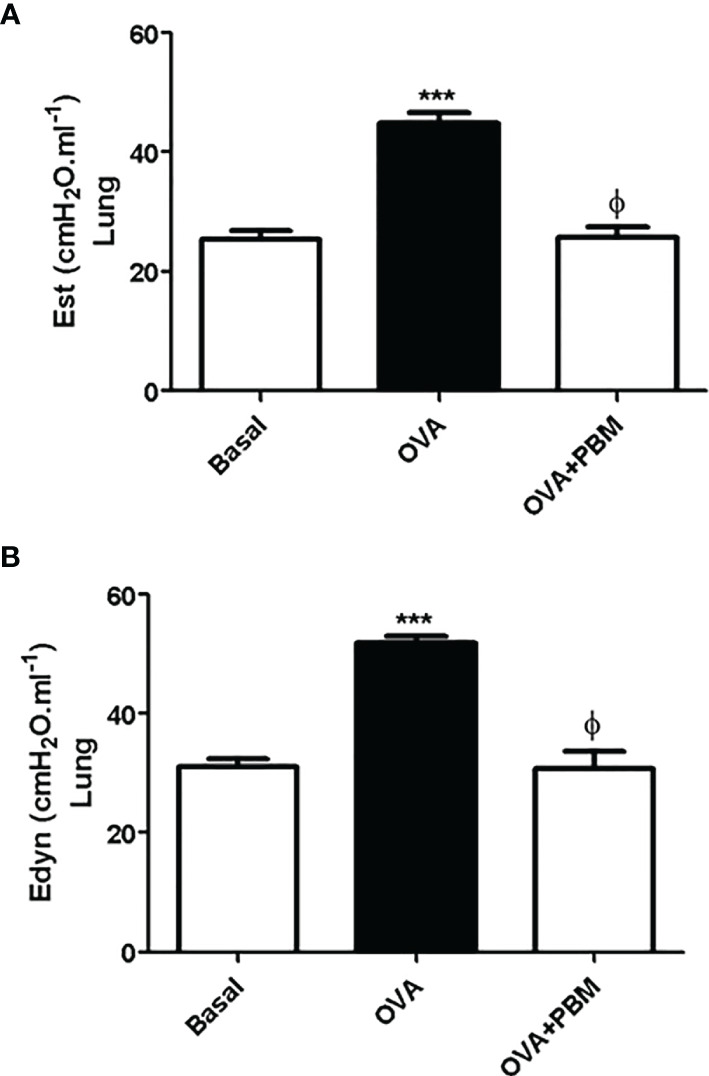
Effect of treatment of photobiomodulation on static **(A)** and dynamic **(B)** elastance in the lung (open chest). Basal group, *n* = 5; OVA group, *n* = 7; PBM group, *n* = 5; OVA+ PBM, *n* = 8. Two independent sets of experiments were carried out. Data are expressed as mean ± SEM. ***p < 0.001 in relation to the Basal group; ^ϕ^ *p* < 0.001 in relation to the OVA group.

**Figure 7:**
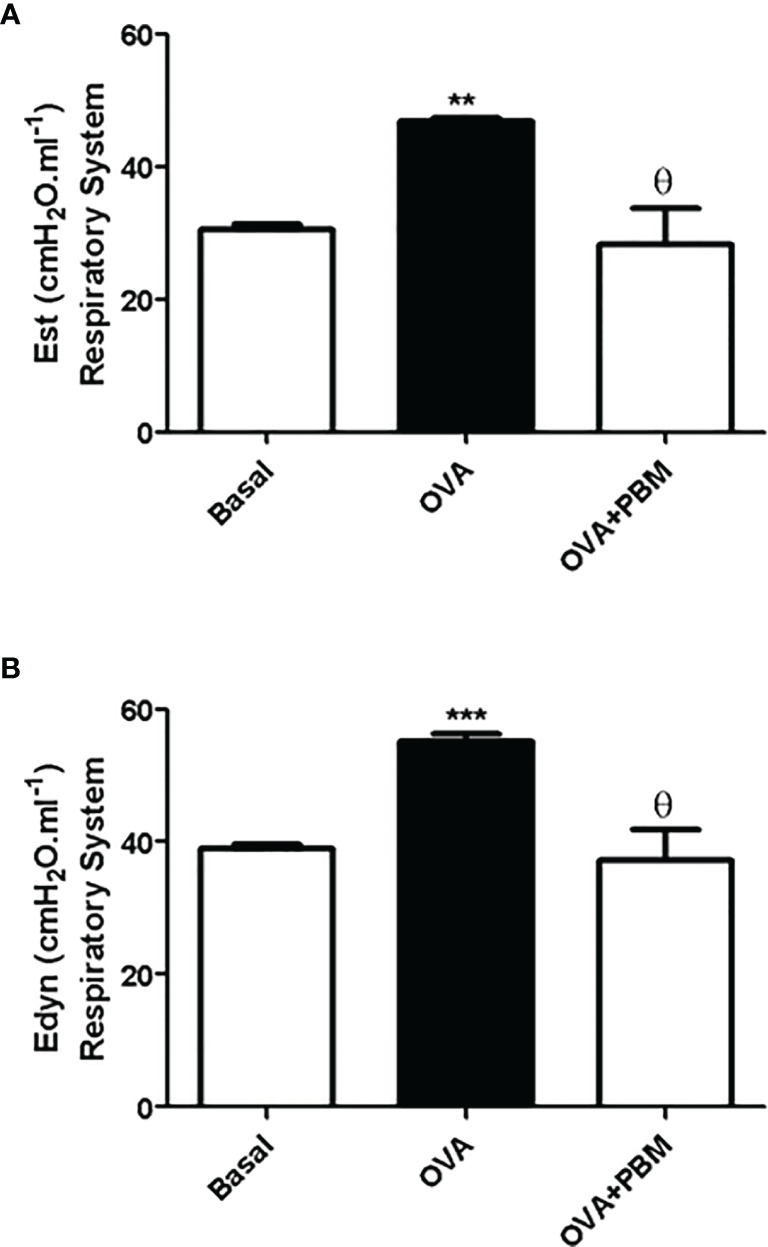
Effect of treatment of photobiomodulation on static **(A)** and dynamic **(B)** elastance in the respiratory system (closed chest). Basal group, n = 5; OVA group, n = 7; PBM group, n = 5; OVA+ PBM, n = 8. Two independent sets of experiments were carried out. Data are expressed as mean ± SEM. **p < 0.01; ***p < 0.001 in relation to the Basal group; Ɵ p < 0.001 in relation to the OVA group.

As shown in **Figures 6** and **7**, the lung and respiratory system elastance values [Est (**Figures 6A** and **7A**) and Edyn (**Figures 6B** and **7B**)] were increased in the OVA group when compared to the Basal group. On the other hand, we observed that the PBM in the OVA group (OVA + PBM) significantly reduced these elastance values, when compared to the OVA group.

### PBM Increased CD4^+^CD25^+^Foxp3^+^ (Treg) and CD4^+^CD25^+^Foxp3^+^IL-10^+^ Cells Percentage in Lung

**Figure 8:**
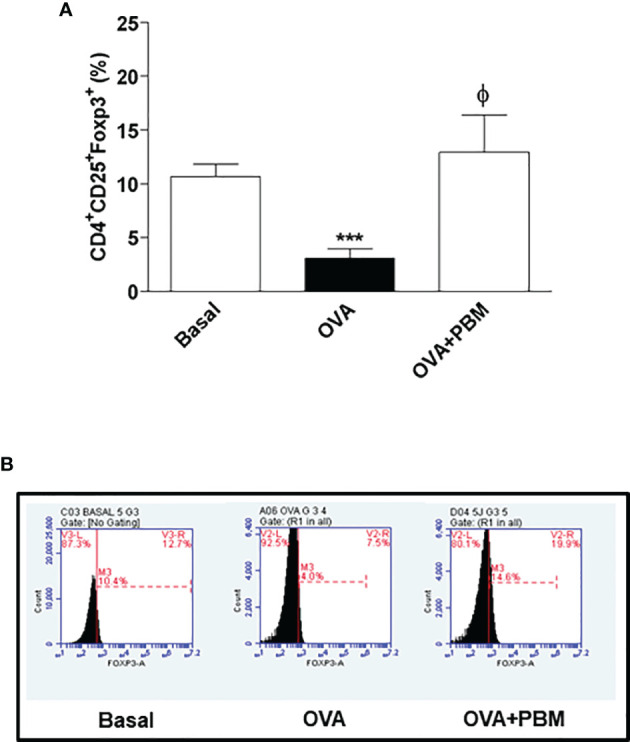
Effect of photobiomodulation on the percentage of CD4+CD25+Foxp3+ Treg cells in BALF. **(A)** Cells were expressed in MFI and live cells were selected and then forward versus side scatter (FSC vs. SSC) gating was used to identify cells of interest based on size and granularity (complexity). The gates strategy was used, where the total cell population in BALF was selected, and then CD4+ T lymphocytes, CD25+ and Foxp3+. Basal group, n = 5; OVA group, n = 7; PBM group, n = 5; OVA+ PBM, n = 8. Two independent sets of experiments were carried out. The histograms represent the all groups evaluated **(B)**. Data represent mean ± standard error (SEM). ***p < 0.001 in relation to the Basal group. ϕ p < 0.001 in relation to the OVA group.

**Figure 9:**
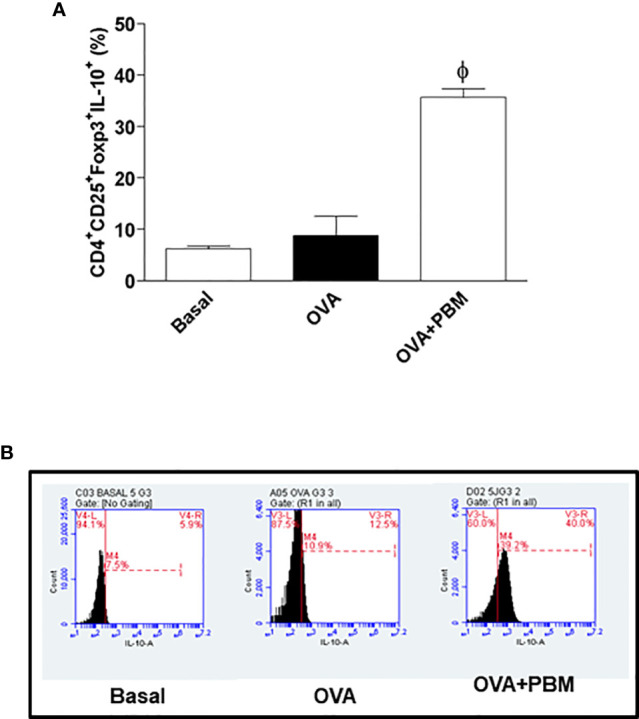
Effect of photobiomodulation on the percentage of CD4+CD25+Foxp3+IL-10+ Treg cells in BALF. **(A)** Cells were expressed in MFI and live cells were selected and then forward versus side scatter (FSC vs. SSC) gating was used to identify cells of interest based on size and granularity (complexity). The gates strategy was used, where the total cell population in BALF was selected, and then CD4+ T lymphocytes, CD25+ ,Foxp3+ and IL-10+. Basal group n=5; OVA group n=7; PBM group n=5; OVA+ PBM n=8. Two independent sets of experiments were carried out. The histograms represent the all groups evaluated **(B)**. Data represent mean ± standard error (SEM). ϕ p<0.001 in relation to the OVA group.

We verified a significant decrease of Treg cells in the lung in the OVA group when compared to the Basal group. When comparing all the asthmatic groups that were submitted to PBM (OVA + PBM), we observed a significant effect on the increase of Treg cells in the lung (**Figure 8A**) when compared to the OVA group. The same increase was verified in **Figure 9A** when evaluated the IL-10 intracellular in Treg cells. The dot plots represent all groups used (**Figures 8B** and **9B**).

## Discussion

As we know, for asthmatic patients, there is an urgent need to develop new anti-inflammatory therapies with immunomodulatory activity to provide alternative strategies for their treatment. In the pathophysiology of asthma, all characteristics of pulmonary inflammation and physiological modulation are the final result of molecular and cellular events involved in sensitization, activation of Th2 cells, and the effector mechanisms of these mediators.

The results of this study showed an increase in the BAL cell profile, in particular an increase in the number of eosinophils. The increased eosinophilic infiltrate in the lung of the asthmatic group is correlated with its growth, development, and increased survival favored by the cytokine IL-5. Eosinophils, once activated, migrate to the site of inflammation and initiate secretion of various inflammatory substances (21). Furthermore, in this experimental model, we observed an increase in macrophages, neutrophils, and lymphocytes, corroborating with literature data (22). On the other hand, we showed that PBM therapy had an anti-inflammatory effect, decreasing the number of inflammatory cells in BAL. We also noticed a significant decrease in the number of eosinophils, the predominant cell in chronic asthma inflammation. In addition, our results demonstrated an increase in the levels of the main pro-inflammatory cytokines found in chronic asthma (IL-4, IL-5, and IL-13) and reduced the anti-inflammatory IL-10. However, when we use a PBM therapy, we observed a decrease in IL-4, IL-5, and IL-13 levels and an increase in the IL-10 levels in BAL. These data agree with those observed previously by our research group, in an experimental model of asthma induced by house dust mite (HDM) when we irradiated animals with laser (14).

In this later stage of the disease, it is possible to observe the airway remodeling process. This stage is characterized by the release of several growth factors and collagen fiber deposition in an attempt to repair the epithelial damage suffered. Previously, we showed greater quantification of collagen fiber deposition around the airway in the allergen-sensitized asthmatic group, a characteristic observed in the chronic phase of the disease, as evidenced in some studies (23). Similar to the data presented in the literature, we found that the release of mediators will trigger injuries and changes in the integrity of the bronchial epithelium, abnormalities in airway muscle tone, changes in mucociliary function, and increase in muscle thickening airway, as well as in its reactive response to stimuli (24, 25).

Cells play an important role in both processes, whether in the inflammatory process or remodeling, and are also directly associated with the obstructive process found in asthma (26, 27). Furthermore, we observed increased mucus production and collagen deposition in the airways in this model of experimental asthma induced by OVA. When we performed PBM therapy, we found a reduction in these pulmonary remodeling parameters.

The beneficial effects of PBM therapy observed in the present study were reinforced by functional measurements, as demonstrated by improved static and dynamic elastance. These results are particularly important, since an increase in static elastance and dynamic elastance observed in the OVA group resembles typical impairment of human asthma (28). Through our results, we can see that in the groups challenged with OVA, there was an increase in lung elastance. It is also worth mentioning that treatment with PBM significantly reduced airway elastance. These data suggest that PBM therapy promotes an improvement in lung function, reducing leukocyte migration to the lung and production of inflammatory mediators in the lung.

Advances have been made to define the mechanisms that control inflammation and induce immune tolerance to specific antigens. Regulatory T cells (Treg) have a suppressive effect on other CD4^+^ T effector cells and may play a role in regulating Th2 function in asthma. It was recently shown that Treg cells can interfere with the development of allergic diseases, including asthma, at different stages such as allergic sensitization, progression to allergic inflammation, bronchial remodeling, hyperresponsiveness, and persistence of the clinical manifestations of the disease. One result of this interaction was that Treg Foxp3 cell could inhibit Th2 cell differentiation directly. Furthermore, the relationship between Foxp3 occurs through an IL-10-dependent mechanism (29). In this sense, recent studies used manipulated mice, where they only had Foxp3 Tregs, but did not have IL-10. Thus, we clearly noted that IL-10 produced by Treg Foxp3 cells was important to suppress allergic lung inflammation in response to allergen challenge.

Regarding these findings, we can suggest that there is an anti-inflammatory potential of the laser, probably *via* the Treg lymphocyte, releasing increased levels of IL-10 to control lung inflammation. In this scenario, the increase found is related to Treg cells, which justifies the high levels of IL-10 in the BALF of animals after PBM therapy, with an effective anti-inflammatory action (30). Similarly, in a study carried out in an experimental model of asthma, laser was able to increase IL-10 levels (31). The literature describes that IL-10 has an important role in controlling the magnitude of pulmonary inflammation, as it regulates the production of IL-4 and IL-5 by Th2 lymphocytes (31), in addition to regulation of mast cell-mediated IgE activation. Other studies carried out in animal models of asthma also report that this cytokine (IL-10) is able to inhibit airway inflammation and hyperreactivity (31). In asthmatic patients, however, IL-10 levels are reduced when compared to healthy individuals, corroborating our results (32).

In this scenario, it is important to draw our attention to the fact that even in mice from the Basal group, IL-10 levels vary a lot, and thus it is possible to find values of IL-10 a little bit higher than those found in most studies. In fact, diverse authors have also found values higher than 50 pg/ml in BALF of mice from the Basal group (33–36). Our results are in accordance with findings in which IL-10 level in BALF of the Basal group reached 1,500 pg/ml (37). Although our results are in agreement with studies, the values of IL-10 found in BALF of Basal groups is truly elevated; however, it does not seem to be a discrepant result since, regardless of IL-10 values in the Basal group, it is important to highlight that the BALF IL-10 level in the Basal group is higher than values of pro-inflammatory mediators found also in the Basal group. This condition in the lung corroborates with authors that describe the imbalance between the IL-10-induced anti-inflammatory response and the pro-inflammatory response in healthy individuals as well as in mice from the Basal group in model allergic asthma. In our point of view, high levels of IL-10 are not interpreted as a problem, but rather show what several manuscripts have already shown; that is, levels of IL-10 in control animals are higher than in sick animals. From this point of view, it would not be possible to study the modulation, which has already been described, between the Th2 and Treg responses. Despite the data from our group, the studies were carried out by different investigators in different experimental models (38, 39). This reinforces our results, since the phenomenon of imbalance between Th2/Treg immune response represented by high levels of IL-10 in the control or Basal group, and low levels in animals in the sick group, was repeated.

Therefore, there is evidence that CD25+Foxp3+ Treg cells play a suppressive role in Th2 immune responses in humans, and similar findings have been described in animals (32, 40). Recent advances in the field of immunology have expanded our knowledge of how adaptive immune responses are regulated, and there is more evidence that Treg cells are an important component of this process, including their participation in the allergic inflammation of asthma (41).

It is important to highlight that our proposal for the use of laser therapy in chronic asthmatic patients was built based on the idea that the photobiomodulator effect of low-level laser can help in conventional pharmacological treatment, so that the dose of corticosteroids can be reduced, and thus it also attenuates the side effects of steroid treatment for chronic asthma. For this reason, it is essential to understand the cellular mechanism of action responsible for the beneficial effect of laser therapy in individuals with chronic asthma, that is, which cellular and molecular targets are directly involved in lung inflammation that are involved in the laser effect. In contrast, the physical principle of laser effect is the interaction of photons with cell receptors. This means that in clinical practice in which laser therapy is applied non-invasively, transcutaneously, it is not possible to determine how much energy is absorbed by lung cells. Unlike pharmacological therapy, it is not possible to measure the amount of energy in the plasma of chronic asthmatics. This condition makes it difficult to characterize the laser dose in relation to asthma symptoms. However, it has been possible to correlate the energy delivered to the patient and the anti-inflammatory effects of the laser. Moreover, some wavelength and irradiation time have been shown to be more effective in the treatment of chronic inflammatory diseases. These dosimetry studies associated with investigation of the photobiomodulator mechanism of action reinforce the possibility of increasing the use of laser therapy as an anti-inflammatory agent in chronic asthmatic individuals.

After all the analysis of the results, we can suggest the effectiveness of the use of PBM therapy. PBM presents significant and effective results in the reduction of eosinophils, production of IL-4, IL-5, and IL-13 in BAL, airway remodeling, and lung function in asthma. Finally, we emphasize the importance of using laser therapy, since we were able to add new evidence, which may contribute to better elucidate the anti-inflammatory effect to be used in an experimental model of asthma induced by OVA.

In conclusion, our results demonstrate the effectiveness of PBM in the regulation of allergic lung inflammation in a chronic experimental asthma model. This therapy with laser reduced cell migration to the lung, levels of cytokine and leukotriene B4, remodeling of airways (mucus and collagen), and pulmonary function. The possible mechanism is the increase in Treg cells that produce IL-10 in the lung; in this sense, this therapy can be used as an immunotherapeutic strategy in the treatment of asthma.

## Data Availability Statement

The raw data supporting the conclusions of this article will be made available by the authors, without undue reservation.

## Ethics Statement

The animal study was reviewed and approved by Nove de Julho University.

## Author Contributions

Conception and design of the study were done by AO and FA. Research was performed by TS, KH, CiA, CrA, and NR-O. Drafting the article was done by AO and RP. MC analyzed the data. Language edit was done by RP and FA. Manuscript revision was done by MC, FA and RP. All authors contributed to the article and approved the submitted version.

## Conflict of Interest

The authors declare that the research was conducted in the absence of any commercial or financial relationships that could be construed as a potential conflict of interest.

## Publisher’s Note

All claims expressed in this article are solely those of the authors and do not necessarily represent those of their affiliated organizations, or those of the publisher, the editors and the reviewers. Any product that may be evaluated in this article, or claim that may be made by its manufacturer, is not guaranteed or endorsed by the publisher.
